# *In Vitro* Evaluations of Cytotoxicity of Eight Antidiabetic Medicinal Plants and Their Effect on GLUT4 Translocation

**DOI:** 10.1155/2013/549345

**Published:** 2013-03-28

**Authors:** Sleman Kadan, Bashar Saad, Yoel Sasson, Hilal Zaid

**Affiliations:** ^1^Qasemi Research Center-Al-Qasemi Academy, P.O. Box 124, 30100 Baqa El-Gharbia, Israel; ^2^Casali Institute for Applied Chemistry, Institute of Chemistry, The Hebrew University of Jerusalem, Givat Ram, 91904 Jerusalem, Israel; ^3^Faculty of Arts and Sciences, Arab American University Jenin, P.O. Box 240, Jenin, Palestine

## Abstract

Despite the enormous achievements in conventional medicine, herbal-based medicines are still a common practice for the treatment of diabetes. *Trigonella foenum-graecum*, *Atriplex halimus*, *Olea europaea*, *Urtica dioica*, *Allium sativum*, *Allium cepa*, *Nigella sativa*, and *Cinnamomum cassia* are strongly recommended in the Greco-Arab and Islamic medicine for the treatment and prevention of diabetes. Cytotoxicity (MTT and LDH assays) of the plant extracts was assessed using cells from the liver hepatocellular carcinoma cell line (HepG2) and cells from the rat L6 muscle cell line. The effects of the plant extracts (50% ethanol in water) on glucose transporter-4 (GLUT4) translocation to the plasma membrane was tested in an ELISA test on L6-GLUT4myc cells. Results obtained indicate that *Cinnamomon cassia* is cytotoxic at concentrations higher than 100 **μ**g/mL, whereas all other tested extracts exhibited cytotoxic effects at concentrations higher than 500 **μ**g/mL. Exposing L6-GLUT4myc muscle cell to extracts from *Trigonella foenum-graecum*, *Urtica dioica*, *Atriplex halimus*, and *Cinnamomum verum* led to a significant gain in GLUT4 on their plasma membranes at noncytotoxic concentrations as measured with MTT assay and the LDH leakage assay. These findings indicate that the observed anti-diabetic properties of these plants are mediated, at least partially, through regulating GLUT4 translocation.

## 1. Introduction

Traditional Greco-Arab and Islamic herbal-based preparations have gained enormous popularity in the Arab world as well as Islamic world over the past three decades. This widespread use and popularity have also brought some concerns and fears over professionalism of traditional healers, quality, efficacy, and safety of the “natural” products available on the market [1]. According to ethnopharmacological surveys there are at least 2,600 plant species in the Middle Eastern region. About 700 of these plants are noted in medieval Greco-Arab and Islamic medical books for their therapeutical use [2]. More than 450 of these plants have continued to be employed in the treatment and prevention of human diseases in most Arab-Islamic countries. Safety assessment of herbal products has often been neglected since prolonged and apparently safe use usually is considered as an evidence of its natural safety. Another very important factor is the belief that these medicines are prepared according to the principles of the Greco-Arab medicine that builds the basis for the modern Western medicine. Therefore, most producers and caregiver institutions of Arab herbal medicines are named after the famous scholars Ibn Sina (Avicenna, 980–1037), Al-Razes (Rhazes, 864–930), and Al-Kindi (Alkindus, 800–873) [3]. However, a prolonged traditional and apparently safe usage is not always a reliable guarantee of safety since it is difficult for traditional practitioners to monitor or to detect delayed effects (e.g., carcinogenicity, mutagenicity), rare adverse effects, and adverse effects arising from long-term use. Adulteration, inappropriate formulation, or lack of understanding of plant and drug interactions or uses might result in life-threatening or lethal side effects. Most reports concerning toxic effects of herbal medicines are associated with hepatotoxicity. Yet, reports of other toxic effects including kidney, nervous system, blood, cardiovascular and dermatologic effects, mutagenicity, and carcinogenicity have also been published in the biomedical literature [3]. Several reasons can cause herbals toxicity, such as, lack of pharmaceutical-level quality control at all stages of production; confusing nomenclature and inaccurate plant identification; variations in levels of active ingredients in different plant parts, plants harvested at different times or stages of development; or the geography, weather, soil, and other conditions specific to the plants growth and development. The complex chemical mixtures of plants and interactions with other herbs, drugs, adulterants, or contaminants; accidental or deliberate unprofessional, unwise, or careless practitioner treatments or recommendations; or incorrect patient use also contribute to safety issues and increase the risk of adverse reactions. Moreover, contamination of herbals with microorganisms, fungal toxins such as aflatoxin, pesticides, heavy metals, and synthetic drugs has been described [4]. Herbal medications are usually mixtures of several ingredients or plants harvested during different seasons and extracted through variable procedures, which makes the identification of both the pharmacologically active and toxic compounds difficult [5]. 

Diabetes is metabolic disease usually caused by a combination of hereditary and environmental factors, which result in hyperglycemia and other classical symptoms, especially polyuria, polydipsia, and polyphagia. Eventually, hyperglycemia leads to serious damage in blood vessels and the nerves as well as blurred vision and irritability. According to the World Health Organization (WHO), the number of people with diabetes will be doubled within less than 30 years. The prevalence of diabetes is the highest in the Middle East, where the number of diabetic subjects reached 15.2 million in 2000. The number of diabetic in the Middle East will almost triple by 2030 (from 15.2 million in 2000 to about 42.6 millions in 2030). This rapid increase is due to population growth, aging, urbanization, increasing prevalence of obesity, and physical inactivity. Diabetes has been recognized by medieval Greco-Arab physicians and its main symptoms were known by the increased thirst, frequent urination, and tiredness. Greco-Arab physicians and practitioners had used series of medicinal plants for treating these combined symptoms. In addition to several instructions for consumption of specific food, mild exercises were recommended. For example, Avicenna (980–1037), a renowned physician of the Golden Ages of the Arab-Islamic civilization, described diabetes in his book *The Canon of Medicine*. He mentioned gangrene and collapse of sexual function as a complication of this disease. Avicenna and other Greco-Arab physicians recommended the use of various medicinal plants for the treatment of diabetes. Fenugreek, walnut, salt bush, olive nettle, garlic, onion, black seed, and cinnamon are just a few of the medicinal plants that are strongly recommended as antidiabetic and antioxidant [6–8]. Bioactive ingredients of some of these plants have been investigated for their efficacy in treating various human diseases. The antidiabetic mechanisms of a plant extract are usually insulin sensitizer, insulin mimics, insulin secretagogues, and inhibitors of intestinal carbohydrate digestion and absorption. Insulin sensitizers include plants that increase glucose uptake and disposal by muscle, fat, and hepatic cells as well as those that regulate hepatic glycogen metabolism. In this category, garlic and onion decrease blood glucose levels by normalizing liver hexokinase and glucose-6-phosphatase activity [9]. Black seed and cinnamon were suggested to have insulin mimetic properties, through enhancing insulin signaling pathway independently of insulin [10].

In the present *in vitro* study we tested the safety of *Trigonella foenum-graecum* (fenugreek), *Atriplex halimus* (salt bush), *Olea europaea* (olive), *Urtica dioica* (nettle), *Allium sativum* (garlic), *Allium cepa* (onion), *Nigella sativa *(black seed), and *Cinnamomon cassia* (cinnamon). Furthermore, the effects of these plant extracts on glucose transporter-4 (GLUT4) translocation to plasma membrane was tested on L6 muscle cells stably expressing myc-tagged GLUT4 (L6-GLUT4myc) using cell-ELISA test. Glucose uptake into skeletal muscle is mediated by the facilitative hexose transporter, GLUT4, a membrane protein that continuously cycles between intracellular stores and the plasma membrane (PM). Insulin primarily promotes the rate of GLUT4 exocytosis and fusion with the PM, a process termed GLUT4 translocation that results in a gain in surface GLUT4 [11]. Results obtained in the present *in vitro* study indicate that 50% ethanol extracts from *Trigonella foenum-graecum*, *Urtica dioica*,* Atriplex halimus*, and *Cinnamomun verum* led to a significant gain in GLUT4 translocation at noncytotoxic concentrations as measured with MTT assay and the LDH leakage assay. These findings indicate that GLUT4 translocation is involved in the observed antidiabetic properties of these medicinal plants.

## 2. Materials and Methods

### 2.1. Materials

Fetal bovine serum, *α*-MEM (modified Eagle's medium), D-MEM standard culture medium, and all other tissue culture reagents used were purchased from biological industries (Beit Haemek, Israel). Horseradish-peroxidase- (HRP-) conjugated goat anti-rabbit antibodies were obtained from Promega (Madison, WI, USA). Polyclonal anti-myc (A-14) and other standard chemicals were purchased from Sigma.

### 2.2. Plant Extract Preparation

*Trigonella foenum-graecum* (seeds), *Atriplex halimus* (leaves and stem), *Olea europaea* (leaves), *Urtica dioica* (leaves and stem), *Allium sativum* (bulb), *Allium cepa* (bulb), *Nigella sativa* (seeds), and *Cinnamomon cassia* (bark) were purchased from Al Alim-Medicinal Herb Center, Zippori, Israel. Fifteen grams of the hand grinded plant material were added to 100 mL of 50% ethanol in double distilled water and heated for 15 minutes at 60°C under stirring. Extract supernatants obtained were passed through a 0.2 *μ*m filter and stored in aliquots at −80°C for further experimental work.

### 2.3. Cell Culture

 Cells from the HepG2 cell line were purchased from ATCC (HB-8065). Cells from the rat L6 muscle cell line, stably expressing myc-tagged GLUT4 (L6-GLUT4myc), being a kind gift from Prof. Amira Klip (The Hospital for Sick Children, Toronto, Canada), were maintained in myoblast monolayer culture. All cells were grown under an atmosphere of 95% air and 5% CO_2_ in *α*-MEM (L6 cells) or D-MEM (HepG2 cells) supplemented with 10% fetal calf serum (FCS), 1 mM L-glutamine, 100 U/mL penicillin, and 0.1 mg/mL streptomycin.

### 2.4. MTT Assay

The MTT assay is based on the protocol described for the first time by Mosmann [12]. The assay was optimized for the cell lines used in the experiments. 3-(4,5-Dimethylthiazol-2-yl)-2,5-diphenyl tetrazolium bromide (MTT) was applied to assess cell viability as described in [13]. Cells (2 × 10^4^/well) were plated in 100 *μ*L of medium/well in 96-well plates and were allowed to attach to the plate for 24 h. Plant extracts were added at increasing concentrations (0–2 mg/mL) for 24 h. The cells medium was replaced with 100 *μ*L fresh medium/well containing 0.5 mg/mL MTT and cultivated for another 4 h darkened in the cells incubator. The supernatant was removed and 100 *μ*L isopropanol/HCl (1 mM HCl in 100% isopropanol) were added per well. The absorbance at 570 nm was measured with microplate reader (Anthos). Two wells per plate without cells served as blank. All experiments were repeated three times in triplicates. The effect of the plants extracts on cell viability was expressed using the following formula:

(1) Percent  viability   =  (A 570 nm  of  plant  extract  treated  sampleA 570 nm  of  nontreated  sample)  ∗100.

### 2.5. Lactate Dehydrogenase Assay (LDH)

LDH, a cytoplasmic enzyme, release is the consequence of cell membrane rupture. Activity of LDH released to the cell culture medium was monitored following the formation of formazan by coupled enzymatic reaction at 500 nm according to the manufacture kit (Promega). Cell membrane rupture was defined as the ratio of LDH activity in the supernatant of treated cells to the LDH activity released in the control cells. HepG2 and L6-GLUT4myc cells (2 × 10^4^/well) were plated in 100 *μ*L of medium/well in 96-well plates and were allowed to attach to the plate for 24 h. After cell attachment (24 h) cells were treated with increasing concentrations of the plant extracts (0–2 mg/mL). The extracellular LDH activity was measured in the medium after 24 h. Therefore, 50 *μ*L from each well was transferred to a new 96 well plate; the enzyme reaction was carried out according to the manufacture kit (CytoTox 96, Promega). All experiments were repeated three times in triplicates. The effect of the plants extracts on cell viability was expressed using the following formula:

(2) Percent  viability =((A 492 nm  of  plant  extract  treated  sampleA 492 nm  of  control)∗100)  −100.

### 2.6. Determination of Surface GLUT4myc

Surface myc tagged GLUT4 was measured in intact, nonpermeabilized cells as previously described [14] using anti-myc antibody followed by secondary antibody conjugated to horseradish peroxidase. Cells grown in 24-well plates for one day followed by addition of the plant extracts for 24 h. Serum starved for 3 h were treated without or with 100 nM insulin for 20 min, washed twice with ice-cold PBS, fixed for 10 min with 3% paraformaldehyde, blocked 10 min with 3% (v/v) goat serum and reacted with polyclonal anti-mycantibody (1 : 200) for 1 h at 4°C, washed 10 times with PBS and reacted with horseradish peroxidase-bound goat anti-rabbit secondary antibody (1 : 1000) for 1 h at 4°C, and washed 15 times with PBS. Cells were then incubated with 1 mL *o*-phenylenediamine dihydrochloride reagent and allowed to develop for 20–30 min in the linear range in the dark at room temperature. The reaction was stopped with 1 mL/well of 3 N HCl. Supernatants were collected and absorbance was measured at 492 nm. Background absorbance obtained in the absence of anti-mycantibody was subtracted from all values.

### 2.7. Statistical Analysis

Error limits cited and error bars plotted represent simple standard deviations of the mean. When comparing different samples, results were considered to be statistically different when *P* < 0.05 (Student's *t*-test for unpaired samples). 

## 3. Results and Discussion

**Figure 1:**
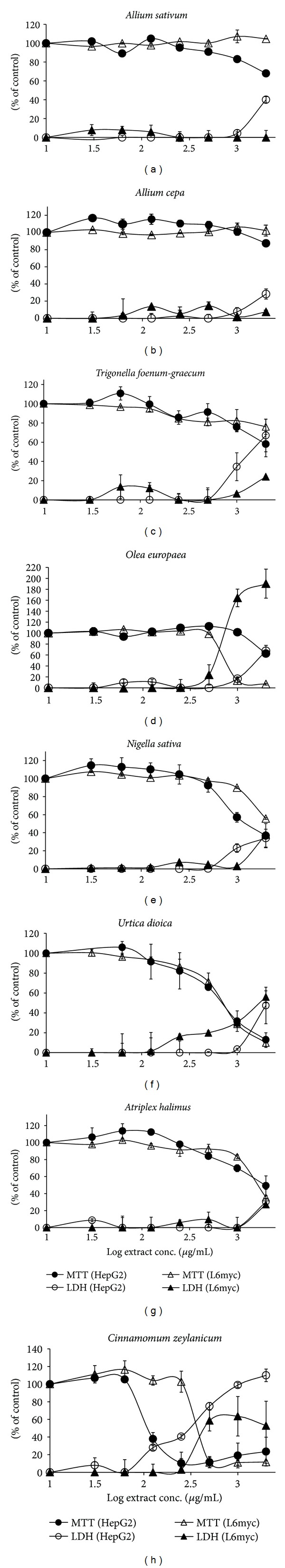
Cytotoxic effects of plant extracts tested on cells from HepG2 and L6myc cell lines. L6-GLUT4myc cells and HepG2 cells were seeded in 96-well plate (20,000 cells/well), exposed to *Allium sativum *(a), *Allium cepa *(b), *Trigonella foenum-graecum *(c), *Olea europaea *(d), *Nigella Sativa* (e), *Urtica pilulifera* (f), *Atriplex halimus* (g), and *Cinnamomum zeylanicum* (h) for 24 h. Cytotoxicity was measured by LDH leakage assay and MTT assay. Each point represents the mean of the data from three independent experiments; bars represent the standard error (S.E) relative to the control. In order to decrease the *y*-axis scale in the LDH presented calculated results, a 100% (control value) was subtracted from all the results shown.

Cytotoxic effects of 50% ethanol/water extracts of *Trigonella foenum-graecum*, *Atriplex halimus*, *Olea europaea*, *Urtica dioica*, *Allium sativum*, *Allium cepa*, *Nigella sativa*, and *Cinnamomon cassia* were evaluated in cells from the human hepatocellular carcinoma (HepG2) and cells from the rat L6 muscle cell line, stably expressing myc-tagged GLUT4 (L6-GLUT4myc), using the MTT assay and the LDH leakage assay. These two tests are widely used in *in vitro* toxicology studies. They are used for the detection of cytotoxic and other negative effects on cell viability following exposure to test materials. In general, in order to increase the reliability of the results obtained and to avoid overestimation or underestimation of the toxicity of the plant extracts, more than one assay should be used to determine cell viability in *in vitro* studies. Therefore, the MTT assay and the LDH leakage assay (Figure 1) were used here. Cells were treated with increasing concentrations of the plant extracts (up to 4 mg/mL) for 24 h. The MTT results were used to determine the EC_50_ values (Table 1). 

The involvement of glucose transporter (GLUT4) in the observed antidiabetic effects of tested eight medicinal plants was evaluated applying the GLUT4 translocation assay. Insulin increases GLUT4 translocation to the surface of myoblasts, where it mediates the increase in glucose uptake [11]. To examine the contribution of the plants extract to GLUT4 localization on the plasma membrane, the extracts were added to the L6-GLUT4myc cells in the presence or absence of insulin, and GLUT4myc translocation to the plasma membrane was tested as described in the Material and Methods section. Herein we will present and discuss results obtained for the eight plants examined.

### 3.1. *Allium sativum* (Garlic) and *Allium cepa* (Onion)

**Figure 2:**
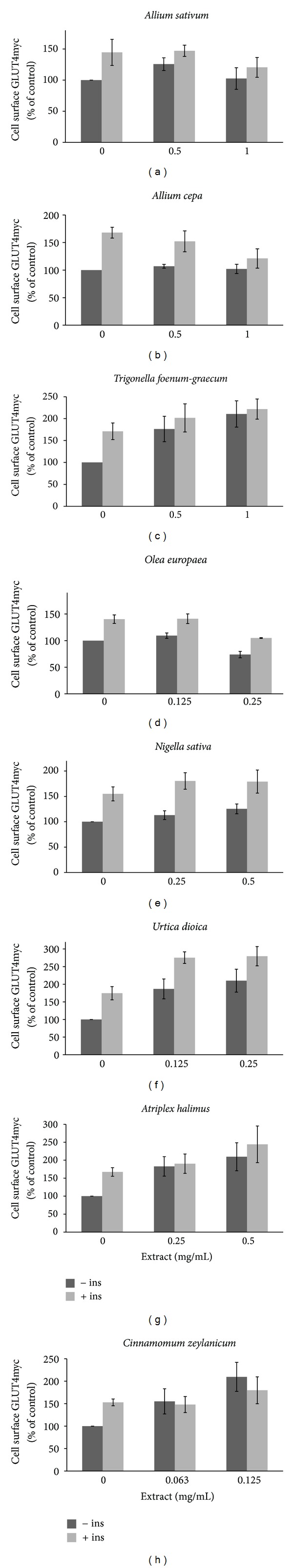
Effect of plant extracts on GLUT4 translocation. L6-GLUT4myc cells were seeded in 24-well plate (100,000 cells/well) and exposed to *Allium sativum *(a), *Allium cepa *(b), *Trigonella foenum-graecum *(c), *Olea europaea *(d), *Nigella Sativa* (e), *Urtica pilulifera* (f), *Atriplex halimus* (G), and *Cinnamomum zeylanicum* (h) for 24 h. Serum depleted cells were treated without or with 100 nM insulin for 20 min at 37°C and surface myc-tagged GLUT4 density was quantified using the antibody coupled colorimetric assay. Shown are the means ± S.E relative to basal nontreated cells from three independent experiments (each has three triplicates).

*Allium sativum* and *Allium cepa* are widely used in the prevention and treatment of various diseases, including but not limited to infections, cancer, and diabetes [15]. Both, the garlic cloves as well as onion bulbs share many similar active compounds (e.g., allyl propyl and diallyl sulfide), decrease blood glucose levels also by normalizing liver hexokinase and glucose-6-phosphatase activities [9], and increase insulin secretion from the pancreas. However, excessive onion and garlic consumption might lead to harmful effects on the body [15]. This observation was confirmed in the present study. As shown in Figure 1(a), no significant reduction in cell viability of HepG2 and L6myc cell lines was seen at any concentration up to 1 mg/mL as assessed with MTT and LDH assays. Reduction of cell viability was seen at very high concentrations, which confirms the description of [15]. Regarding their reported hypoglycemic properties, our results indicate that the antidiabetic properties of these two plants are not mediated through GLUT4 translocation to the PM. As shown in Figures 2(a) and 2(b), garlic and onion extracts did not alter the amount of surface-exposed GLUT4myc in the basal (unstimulated) state and even had slightly decreased GLUT4myc translocation to the plasma membrane in the insulin stimulated state when cells were treated with 1 mg/mL of the plants extracts.

### 3.2. *Trigonella foenum-graecum* (Fenugreek)

*Trigonella foenum-graecum* has been used to treat diabetes and sore throats and in poultices used to treat sores and abscesses. Recent investigations into the medicinal properties of fenugreek suggest its importance not only as a preventive for chronic diseases such as diabetes, but also for enhancing normal physiological processes, especially with respect to athletic performance. Fenugreek seeds are very rich in dietary fibers that modulate delaying the absorption of sugar and cholesterol from the intestines, thus protecting against diabetes, heart disease, and obesity [16].

Clinical and experimental studies show positive effect of the fenugreek seeds in the metabolism of glucose in the body. Fenugreek seeds contain a gel-like soluble fiber which combines with bile acid and lowers triglyceride and LDL cholesterol levels. To maximize their medicinal effect, fenugreek seeds are chopped finely and served as a flavorful or soaked in water for overnight. The nicotinic acid, alkaloid trigonelline, and coumarin contained by defatted section of the seed of fenugreek prove to be the responsible active ingredient for its antidiabetic properties [17, 18]. In clinical trials, low doses had no significant effect on fasting blood glucose (FBG) levels of diabetic subjects. However, higher dose (100 mg/kg body weight) of defatted seed powder for 10 days improved FBG values. Several active ingredients were purified from fenugreek seeds. Some of them isolated from fenugreek seeds (e.g., trigonelline and nicotinic, GII) have antidiabetic properties. Treatment of the moderately diabetic rabbits with a novel active ingredient named GII (100 mg/kg body weight for 3 weeks) reduced fasting blood glucose to nearly normal [19]. To assess the functional relevance of *Trigonella foenum-graecum* extract to GLUT4 traffic, we explored the effects of plant ethanol/water (50%, 50%) extract on GLUT4myc translocation to the PM. GLUT4myc translocation to the cell surface was almost doubled in the presence of 1 mg/mL of *Trigonella foenum-graecum* extract. Insulin stimulated GLUT4myc translocation was slightly increased from 170% in the nontreated cells to about 200% and 230% in the cells treated with 0.5 and 1 mg/mL extract, respectively (Figure 2(c)). Results obtained here indicate that *Trigonella foenum-graecum* extract led to a significant gain in GLUT4 on L6 plasma membrane at noncytotoxic concentrations (up to 1 mg/mL) as measured with MTT assay and the LDH leakage assay. These findings indicate that antidiabetic properties of *Trigonella foenum-graecum *extract are mediated, at least partially, through GLUT4 translocation. In addition to its beneficial antidiabetic effects, no cytotoxic effects were seen with fenugreek up to relatively high concentrations of 1.7 mg/mL (Figure 1(c)). The EC_50_ value of Fenugreek was found to be higher than 2 mg/mL for the two cell lines tested (Table 1). 

### 3.3. *Olea europaea* (Olive Leaf)

*Olea europaea* is one of the most commonly known medicinal plants that have been used for centuries within Greek countries, Arab countries, and others. The major active compounds of olive leaf are oleuropeoside, hydroxytyrosol, tyrosol, caffeic acid, p-coumaric acid, vanillic acid, vanillin, oleuropein, luteolin, diosmetin, rutin, verbascoside, luteolin-7-glucoside, apigenin-7-glucoside, and diosmetin-7-glucoside [20]. Oleuropein and phenolics disclosed distinct hypoglycaemic effects (at a dose of 16 mg/kg, together with hypotensive and hypolipidemic properties). They were reported to posses antioxidant capacity as well as antimicrobial [21]. Various beneficial effects are attributed to olive leaf extracts. Clinical evidence has proven the blood pressure lowering effects of olive leaf extract [22] as well as antiaging, antioxidant immunostimulatory and antibiotic (antibacterial, antifungal, and anti-inflammatory) effects. Said et al., 2008 [23] used powdered olive leaf (in a mixture with *Juglans regia*,* Urtica dioica*, and *Atriplex halimus*) and reported that this mixture decreases glucose absorption from the intestine and lowers blood glucose levels in rats and diabetic subjects. No significant effects on the rate of GLUT4 translocation were seen here when cells treated with 125 *μ*g/mL extract. Interestingly, however, exposing L6-GLUTmyc cells to 250 *μ*g/mL significantly decreased GLUT4 translocation to the PM from 100% to 80% in the basal state (without insulin stimulation) and from 150% to 105% in insulin stimulated cells (Figure 2(d) and Table 1). It seems that the above mentioned antidiabetic effects of olive extracts are mediated by distinct mechanisms than GLUT4 translocation system. In addition, no cytotoxic effects were observed with concentrations up to 0.8 mg/mL as measured with MTT and LDH assays (Figure 1(d) and Table 1).

### 3.4. *Nigella sativa* (Black Seed)

*Nigella sativa* is one of the most commonly used medicinal plants that have been used for centuries as a spice as well as a protective and curative remedy for numerous diseases. Potential toxicity of the fixed oil of the seeds was investigated in mice and rats through determination of LD_50_ values and examination of possible biochemical, hematological and histopathological changes. LD_50_ values, obtained by single doses (acute toxicity) in mice, were 28.8 mL/kg body with oral administration and 2.06 mL/kg body with intraperitoneal administration. Chronic toxicity was studied in rats treated daily with an oral dose of 2 mL/kg body wt for 12 weeks. Changes in key hepatic enzymes levels, including ALT, AST, and GSH, and histopathological modifications (heart, liver, kidneys, and pancreas) were not observed in rats treated with *Nigella sativa* oil after 12 weeks of treatment. Negative effects of *Nigella sativa *were reported by Khader et al., (2007) who tested toxicological properties and potential antimutagenic effects of *Nigella sativa* aqueous extracts using primary rat hepatocyte cultures against N-methyl-N-nitro-N-nitrosoguanidine (MNNG). They found that *Nigella sativa* led to a significant increase of chromosomal aberrations in the case of pretreatment [24]. Thymoquinone, dithymquinone, thymohydroquinone, and thymol, are the main active compounds responsible for the therapeutic effects of *Nigella sativa* seeds. Many scientific reports addressed the antidiabetic effects of plant mixtures containing *Nigella sativa*. These studies revealed that the blood glucose lowering effect was due to the inhibition of hepatic gluconeogenesis. For instance, an aqueous extract of a plant mixture containing *Nigella sativa* was found to lower the blood glucose level significantly after oral administration [25]. Furthermore, intraperitoneal administration of *Nigella sativa* seed's oil produced a significant hypoglycemic effect in normal and alloxan-induced diabetic rabbits. Similar results were seen in rats treated with plants mixture including *Nigella sativa* [26]. Another study with Streptozotocin- and Nicotinamide-induced diabetes mellitus in hamsters revealed that four weeks of treatment with *Nigella sativa* oil result in significant decrease in blood glucose level together with significant increase in serum albumin level [27]. These findings indicate that the hypoglycemic effect of *Nigella sativa* oil is, at least partially, mediated by a stimulation of beta cells coincident with an increase in serum insulin level and possess insulinotropic properties in type II like model. In another study, the hypoglycemic effect of *Nigella sativa* was supposed to be mediated by extrapancreatic actions rather than by stimulated insulin release [28, 29]. Results obtained here indicate that 50% ethanol extracts of 0.25 and 0.5 mg/mL *Nigella sativa* did not affect significantly the GLUT4 translocation in the presence of insulin and slightly increased it (about 20%) in the basal state (Figure 2(e)). Figure 1(a) depicts the MTT and LDH assays results of the treatment of HepG2 and L6myc cells with *Nigella sativa* extract, which was found to be safe up to concentrations of 500 *μ*g/mL. The EC_50_ values for this extract were seen at 1470 *μ*g/mL and higher than 2000 *μ*g/mL for HepG2 and L6myc cell lines, respectively (Table 1). Taken together, the data emerged from these studies indicate that *Nigella sativa* is not toxic, as evidenced by high LD_50_ values, hepatic enzymes stability, and organ integrity, suggesting a wide margin of safety for the therapeutic doses of *Nigella sativa* extracts.

### 3.5. *Urtica dioica* (Nettle)

Its Leaf has a long history as an herbal remedy and nutritious addition to the diet. Nettle leaves are a rich source of essential amino acids, ascorbic acid, several mineral elements and vitamins, such as iron, provitamin A and vitamin C [30]. Nettle is believed to be anticarcinogenic, antiulcer, antioxidant, anti-inflammatory, immune suppressive, and antirheumatoid [31]. There is also evidence that nettle extracts possess hypoglycaemic properties and improve glucose tolerance [23]. Consistently, exposing L6-GLUT4myc cells to 125 and 250 *μ*g/mL of nettle extracts almost doubled GLUT4 translocation to the PM in the basal state and increased about 1.6 fold in the insulin stimulated state (Figure 2(f) and Table 1). Nettle extract was safe to use in the cell lines tested up to 500 *μ*g/mL in L6 and in HepG2 cell lines (Figure 1(f) and Table 1).

### 3.6. *Atriplex halimus* (Saltbush)

*Atriplex halimus* is well known and extensively used to treat diabetes especially in the Middle East. An animal model for diabetogenesis and obesity proved that this plant is an extremely effective antidiabetic herb and shows an insulin potentiating effect. Sand rats were fed diets composed of standard laboratory animal chow with or without *Atriplex halimus*. Saltbush lowered the blood glucose levels and enhanced insulin secretion in animals fed with it [32]. Said et al., showed that saltbush (in a mixture with the leaves of *Juglans regia*,* Urtica dioica*, and *Olea europaea*) is effective in lowering blood glucose levels in diabetic patients. In addition, *in vitro* experiments showed that the mixture facilitates glucose entry into yeast cells during anaerobic fermentation. This observation was attributed to an effect of *Atriplex halimus *content in the mixture [23]. Our results support the previous reported studies and shows that the main antidiabetic mechanism of the saltbush is increasing GLUT4 translocation to the plasma membrane, thereby increasing glucose uptake to the muscle, liver, and fat cells. Exposing L6-GLUT4myc cells to 0.25 and 0.5 mg/mL saltbush extracts almost doubled GLUT4 translocation in the basal state. When treated with insulin, GLUT4 translocation was not altered in the presence of 0.25 mg/mL Saltbush extract but was increased from 160% to 230% in the presence of 0.5 mg/mL of the plant extract (Table 1 and Figure 2(g)). Saltbush seems to be extremely safe for intake as its EC_50_ was about 2 mg/mL (Table 1 and Figure 1(g)).

### 3.7. *Cinnamomun verum* (Cinnamon)

*Cinnamomun verum* possesses antioxidant properties that help to reduce the damaging effects of diabetes [33]. Cinnamon is also believed to increase both insulin secretion and the body's cells' sensitivity to insulin via its active ingredient, methyl hydroxy chalcone polymer (MHCP). These two ways of action improve the efficiency of insulin and increase the conversion of consumption of glucose. Results obtained here demonstrate that GLUT4myc translocation to the cell surface was increased by 1.5- and 2-fold in the basal state in the presence of 62 and 125 *μ*g/mL of cinnamon extract, respectively. Insulin stimulated GLUT4myc translocation to the PM was not affected when the L6-GLUT4myc cells were treated with 62 *μ*g/mL, but was slightly increased when treated with 125 *μ*g/mL (Figure 2(h) and Table 1). Results obtained here illustrate efficacy of *Cinnamomun verum* in enhancing GLUT4 translocation to the PM. Cytotoxic effects of *Cinnamomun verum* extracts were seen at concentrations higher than 100 *μ*g/mL for HepG2 cells and higher than 250 *μ*g/mL for L6myc cells as assessed with MTT and LDH assays (Figure 1(h) and Table 1). The EC_50_ of *Cinnamomun verum* was around 100 *μ*g/mL for HepG2 cells and around 390 *μ*g/mL for L6myc cells (Table 1). This cytotoxic effects must be taken into consideration when applying *Cinnamomun verum* as therapeutic or prophylactic agent.

## 4. Conclusions

*Trigonella foenum-graecum*, *Atriplex halimus*, *Olea europaea*,* Urtica dioica*, *Allium sativum*, *Allium cepa*, *Nigella sativa*, and *Cinnamomon cassia* are widely used in the Greco-Arab and Islamic medicine for their antidiabetic and antioxidant properties. Safety assessment of herbal products has often been neglected since prolonged and apparently safe use is usually considered as an evidence of its nature's source. Results obtained here indicate that *Cinnamomon cassia* is cytotoxic at concentrations higher than 100 *μ*g/mL, whereas the other extracts exhibited cytototoxic effects at concentrations higher than 500 *μ*g/mL. *Trigonella foenum-graecum*, *Urtica dioica*, *Atriplex halimus* and *Cinnamomun verum* induced a significant gain in GLUT4 translocation in muscle cells PM at noncytotoxic concentrations. These findings indicate that antidiabetic properties of these medicinal plants are mediated, at least partially, through mediating GLUT4 translocation to the PM.

## Figures and Tables

**Table 1:** GLUT4 translocation and EC_50_ of plant extracts in cells from HepG2 and L6myc cell lines. Data given represent the mean ± SEM from three independent experiments carried out in triplicates.

Plant name	Part used	Cell type	EC_50_ (mg/mL)	Cell surface* GLUT4myc
*Allium sativum *	Bulb	L6myc	>2	−
		HepG2	>2	
*Allium cepa *	Bulb	L6myc	>2	−
		HepG2	>2	
*Trigonella foenum *	Seeds	L6myc	>2	++
		HepG2	>2	
*Olea europaea *	Leaves	L6myc	0.79 ± 0.036	− −
		HepG2	>2	
*Nigella sativa *	Seeds	L6myc	>2	+
		HepG2	1.47 ± 0.49	
*Urtica dioica *	Leaves and stem	L6myc	0.73 ± 0.09	++
		HepG2	0.76 ± 0.13	
*Atriplex halimus *	Leaves and stem	L6myc	1.72 ± 0.22	++
		HepG2	0.8 ± 0.3	
*Cinnamomon cassia *	Bark	L6myc	0.39 ± 0. 01	+
		HepG2	0.12 ± 0. 01	

*Gain in GLUT4 on plasma membranes at noncytotoxic concentrations, (−) no effect, (− −) decreased GLUT4 translocation, (+) slightly increased the GLUT4 translocation, (++) high GLUT4 translocation.
